# Domestication of Plants of *Ugni molinae* Turcz (Myrtaceae) Interferes in the Biology of *Chilesia rudis* (Lepidoptera: Erebidae) Larvae

**DOI:** 10.3390/molecules26072063

**Published:** 2021-04-03

**Authors:** Manuel Chacón-Fuentes, Leonardo Bardehle, Ivette Seguel, Fernanda Rubilar, Daniel Martínez-Cisterna, Andrés Quiroz

**Affiliations:** 1 Laboratorio de Química Ecológica, Departamento de Ciencias Químicas y Recursos Naturales, Universidad de La Frontera, Av. Francisco Salazar 01145, Casilla 54-D, Temuco 4811230, Chile; leonardo.bardehle@ufrontera.cl (L.B.); frubilarv2013@alu.uct.cl (F.R.); d.martinez11@ufromail.cl (D.M.-C.); 2 Centro de Investigación Biotecnológica Aplicada al Medio Ambiente (CIBAMA), Universidad de La Frontera, Av. Francisco Salazar 01145, Casilla 54-D, Temuco 4811230, Chile; 3 Centro de Fruticultura, Facultad de Ciencias Agropecuarias y Forestales, Universidad de La Frontera, Casilla 54-D, Temuco 4811230, Chile; 4 Innovalimentos SPA, Gabriela Mistral 02311, Temuco 4780000, Chile; ivseguel@gmail.com; 5 Carrera de Agronomía, Facultad de Recursos Naturales, Departamento de Ciencias Agropecuarias y Acuícolas, Universidad Católica de Temuco, Manuel Montt 56, Casilla 15-D, Temuco 4810302, Chile; 6 Programa de Doctorado en Ciencias de Recursos Naturales, Universidad de La Frontera, Av. Francisco Salazar 01145, Casilla 54-D, Temuco 4811230, Chile

**Keywords:** phenolic compounds, HPLC, chemical defense, quercetin, kaempferol, myrcetin

## Abstract

In terms of the domestication process in murtilla, studies have found changes in the concentration of phenolic compounds, with reduction of chemical defense of plants, depending on the change in the feeding behavior of insects. Thus, we hypothesized that the domestication of *Ugni molinae* decreases the content of phenolic compounds and modifies the feeding preference of *Chilesia rudis* larvae. Leaves of three parental ecotypes and four cultivated ecotypes were used in preference experiments to evaluate the mass gain and leaves consumption of larvae. Phenolic extracts from leaves of *U. molinae* were analyzed by HPLC. Identified compounds were incorporated in an artificial diet to assess their effect on mass gain, consumption, and survival of the larvae. The presence of phenolic compounds in bodies and feces was also evaluated. In terms of choice assays, larvae preferred parental ecotypes. Regarding compounds, vanillin was the most varied between the ecotypes in leaves. However, plant domestication did not show a reduction in phenolic compound concentration of the ecotypes studied. Furthermore, there was no clear relation between phenolic compounds and the performance of *C. rudis* larvae. Whether this was because of sequestration of some compounds by larvae is unknown. Finally, results of this study could also suggest that studied phenolic compounds have no role in the *C. rudis* larvae resistance in this stage of murtilla domestication process.

## 1. Introduction

Plant domestication has been considered one of the most relevant phenomena in human history due to the capacity for crop cultivation [1,2]. In this framework, cultivated crops have been changing in order to provide anthropogenic necessities, generating differences in their traits in relation to their ancestors that were subjected to a different set of selective pressures. For instance, the most commonly modified organs in terms of human selection have been roots, stems, leaves, flowers, and fruits [3,4]. Furthermore, reductions in chemical defenses in cultivated plants have been related to increased pests or decreased attraction to predators of said pests in relation to parental ecotypes [5,6,7,8]. A native species from Chile, murtilla (*Ugni molinae* Turcz), was cultivated around 25 years ago. Murtilla has been studied because of its potential as an antioxidant source [9,10,11,12,13] and as a plant model in the domestication process [14] related to insect–plant interactions [15]. In general, chemical defenses in plants can be modified by domestication. For example, the authors of [3] reported that breeding in cranberry plants has compromised the defense decreasing the concentration of a quercetin derived, the quercetin-3-*α*-arabinopyranoside from ≈10 mg/g of dry mass in McFarlin cultivar to ≈3 mg/g of dry mass in their cultivated counterpart “NJS98-23”. In particular, studies have continuously related the domestication process in murtilla plants with a reduction of defensive compounds such as flavonoids and an increase of insect pests [15,16,17,18]. Moreover, phenolic compounds (PCs) have been reported as one of the main compounds involved in the feeding behavior of insects [15,16,17,18]. For instance, our previous study reported that the domestication degree, analyzed as a source of variation, significantly affected the PC concentration in leaves of wild and cultivated murtilla [17]. The PC content in leaves of *U. molinae* was reduced from 80% to 70% when the plant was cultivated.

The directionality in murtilla selection was focused mainly on improving yield and production [19,20]. As we reported earlier, murtilla domestication negatively affected PC concentration, and specifically the concentrations of rutin, quercetin, quercetin glucoside, and kaempferol were significantly reduced in cultivated plants [15]. In addition, that report showed that the damage index (DI) of insects was significantly higher in cultivated murtilla ecotype 19-1 of (1.5 DI) than in the respective wild counterpart (0.5 DI). Our previous results suggest that damage by insect pests was negatively correlated with PC concentration and it was tied to cultivated lines having lower PC concentration. The same study reported an increase of the relative abundance of insects from 13.51% in wild plants to 86.48% in cultivated ecotypes [17].

All of the previous studies showed that the chemical defense of murtilla has been reduced by domestication, reporting an increase of insects and damage. In this framework, one insect that has been reported in murtilla is *Chilesia rudis* Butler (Lepidoptera: Erebidae). The first report of this insect in murtilla was in 2009, with the larvae of this insect being associated with both cultivated and wild murtilla [16,21]. This insect in its larval stage is a severe and abundant polyphagus defoliator using *U. molinae* as its host [21]. 

The role of phenolic compounds in the feeding behavior of insects has also been studied [22,23]. However, the role of PCs in the feeding of *C. rudis* has not been evaluated yet, but the consumption of wild and cultivated *U. molinae* leaves by their larvae has been reported [17]. Our previous report showed that *C. rudis* larvae consumption of murtilla increased significantly from wild to cultivated ecotypes (3 cm^2^ to 7 cm^2^) in no-choice assays. In addition, the same study showed a difference in the larvae’s consumption in a choice assay, with an increase in the consumed area of the leaf from wild to cultivated plants (1 to 4 cm^2^). To date, the total of data reported in relation to PCs identified in ancestor wild and cultivated ecotypes of *U. molinae* correspond to a comparison between wild material (obtained from the original wild locations) and their cultivated clones at the local experimental station of the Instituto de Investigaciones Agropecuarias (INIA) Tranapuente, Región de La Araucanía, Chile. This is the first report analyzing the effect of domestication, comparing parental ecotypes material (corresponding to the cultivated ecotypes reported in [15]) with new cultivated ecotypes (offspring) obtained through breeding of the parental ecotypes. Hence, we hypothesized that domestication of *U. molinae* decreases the content of phenolic compounds and modifies the feeding preference of *C. rudis* larvae.

## 2. Results

### 2.1. No-Choice Assay

**Figure 1:**
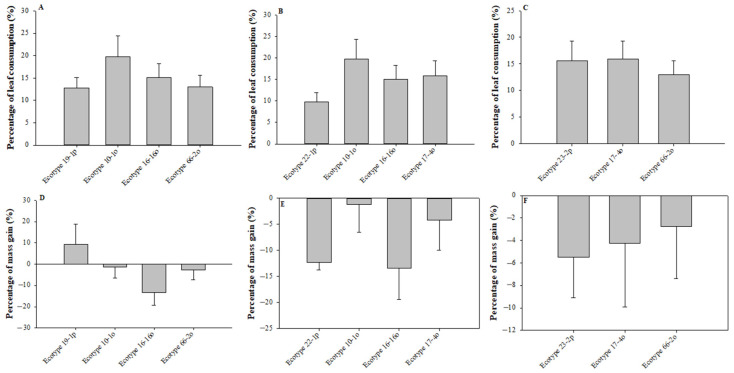
(**A**) Leaf consumption per murtilla ecotype by *C. rudis* larvae comparing ecotype 19-1 vs. their offspring in no-choice assay. (**B**) Leaf consumption per murtilla ecotype by *C. rudis* larvae comparing ecotype 22-1 vs. their offspring in no-choice assay. (**C**) Leaf consumption per murtilla ecotype by *C. rudis* larvae comparing ecotype 23.2 vs. their offspring in no-choice assay. (**D**) Mass gain per murtilla ecotype in *C. rudis* larvae comparing ecotype 19-1 vs. their offspring in no-choice assay. (**E**) Mass gain per murtilla ecotype in *C. rudis* larvae comparing ecotype 22-1 vs. their offspring in no-choice assay. (**F**) Mass gain per murtilla ecotype in *C. rudis* larvae comparing ecotype 23.2 vs. their offspring in no-choice assay. No letters means no significant difference, and letters indicate significance according to ANOVA and Tukey’s test (*p* ≤ 0.05).

Parental and cultivated *U. molinae* leaves did not affect the consumption behavior of *C. rudis* larvae (F_2,49_ = 0.594, *p* = 0.733) (Figure 1A,B). When the parental ecotype 23-2 was compared with their respective cultivated counterpart, we found no differences (Figure 1C). In addition, a higher numeric trend was observed in ecotype 10-1o with a consumption of 19.79 ± 4.60%. Finally, as shown in Figure 1D–F, no significant differences were found for each comparison between parental and cultivated ecotype.

### 2.2. Choice Assay

Choice bioassays showed that consumption and mass gain were significant dependent on the ecotype (Table 1). Moreover, the effect of the ecotypes on consumption (F_11,108_ = 2.503, *p* ≤ 0.001) and mass gain (F_11,108_ = 2.311, *p* = 0.014) on the performance of *C. rudis* larvae was significant. Table 1 shows that *C. rudis* preferred parent ecotype leaves over cultivated leaves, and parental ecotypes 19-1p and 22-1p were more consumed than their offspring 10-1o and 16-16o (except 19-1p × 10-1o). However, larvae chose cultivated leaves over parental ecotypes leaves when the parental ecotype 23-2p was involved with 66-2o ecotype. Moreover, three situations were observed in relation to the larval mass variation: (1) individuals that showed a large mass decrease but chose the parental ecotypes (19-1p vs. 10-1o and 19-1p vs. 16-16o), (2) individuals that showed little mass loss and chose parental ecotypes (19-1p vs. 66-2o, 22-1p vs. 10-1o, and 22-1p vs. 16-16o), and (3) individuals that showed large mass loss and chose cultivated ecotypes (22-1p vs. 17-4o, 23-2p vs. 17-4o, and 23-2p vs. 66-2o). These results indicate that parental ecotype 19-1p and cultivated ecotype 17-4o were responsible for the largest larval mass decreases. In addition, the consumption per ecotype in each comparison was evaluated. Table 1 shows a general preference for parental ecotypes 19-1p and 22-1p, supporting the results observed in Figure 1D–F; similarly, ecotype 23-2p, when compared with cultivated ecotype 17-4o, indicated a preference of *C. rudis* for cultivated leaves. In general, the results shown in Table 1 indicate that parental ecotype plants were more preferred than the respective cultivated ecotypes, and significant differences were seen for the parental ecotypes/cultivated combinations 19-1p and 16-16o, 19-1p and 66-2o, 22-1p and 10-1o, and 22-1p and 16-16o.

### 2.3. Phenolic Compound Extraction

The concentration of PC in *U. molinae* leaves was significantly influenced by ecotype (F_6,124_ = 38.55, *p* ≤ 0.001) and PC (F_8,124_ = 129.15, *p* ≤ 0.001) (Table 2). Furthermore, a significant interaction between ecotypes and PC (F_48,124_ = 2.26, *p* ≤ 0.001) is shown, indicating a synergistic effect of these factors. The results in Table 2 indicate that the parental ecotype 22-1p produced the highest amount of PC (2027.3 ± 79.57 µg/g) and the parental ecotype 23-2p showed the lowest total amount of PC (132.4 ± 7.88 µg/g). Vanillin was the major compound found in *U. molinae* leaves in each parental ecotype and cultivated ecotype. In addition, vanillin, caffeic acid, catechin, and quercetin showed higher amounts of compound in the parental ecotype 22-1p than the other parental ecotypes and cultivated ecotypes. In general, all cultivated ecotypes showed a lower concentration of each PC (except for caffeic acid, catechin, and quercetin) than the common parental ecotype 22-1p.

### 2.4. Dose-Response Experiment

**Figure 2:**
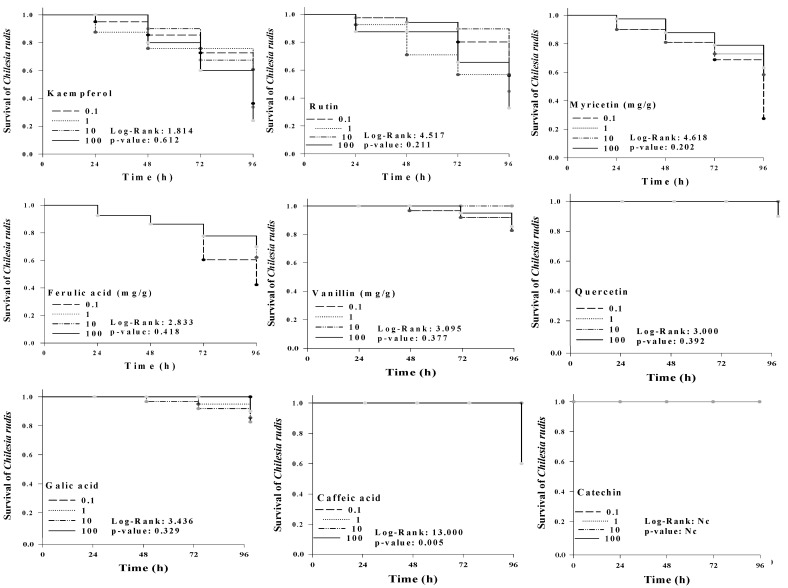
Kaplan-Meier survival curves of *C. rudis* in relation to compounds, concentration, and time according to log-rank analysis (*p* ≤ 0.05).

When PC and the several doses were compared with their respective controls, we found a general decrease in consumption percentage by *C. rudis*. However, a significant increase in the percentage of consumption of quercetin in 0.1 mg/g and rutin in 100 mg/g of diet treatment at 48 h was observed. Vanillin and gallic acid elicited a decrease of diet consumption independently of dose and evaluation period. Kaempferol, caffeic, and ferulic acid elicited a decrease of diet consumption after 48 h, and catechin after 72 h. Myricetin showed low activity, and quercetin elicited a decrease of diet consumption at 24, 72, and 96 h for all doses (Table 3). Interestingly, diet supplemented with PC negatively affected the weight of *C. rudis* larvae (Table 4). This mass variation was more evident after 72 h, and the maximum decrease in larval mass was observed at 96 h for all doses. Gallic and ferulic acid elicited that response for all times and doses, and caffeic acid was similar except at 24 h at a dose of 0.1 mg/g. Furthermore, Student’s *t*-test analysis indicated that myricetin in 1, 10, and 100 mg/g of diet at 24 h and rutin in 0.1, 1, 10, and 100 mg/g were significantly higher than the control, showing an increased mass gain in *C. rudis*. Regression analysis shown in Table 5 indicates for *C. rudis* leaves consumption that independent variable “hours” was influential in the model (except for rutin). In addition, for the mass gain we observed the opposite, wherein “concentration” was more influent than “hours” (except for caffeic acid). In Figure 2, the Kaplan–Meier survival curves for *C. rudis* larvae show that kaempferol, rutin, ferulic acid, myricetin, vanillin, quercetin, and gallic acid were not significant at 0.1, 1, 10, and 100 mg/g of diet. However, caffeic acid showed a significant reduction in survival rate for 100 mg/g of diet at 96 h, decreasing to 90% of survival in relation to other concentrations. Finally, no mortality was observed for catechin, with 100% of survival at 96 h.

### 2.5. Presence of Phenolic Compounds in Bodies and Feces

Compounds (F_9,80_ = 5939.2, *p* ≤ 0.001), concentrations (F_3,80_ = 19,733.6, *p* ≤ 0.001), and their interaction (F_27,80_ = 1770.0, *p* ≤ 0.001) influenced the PC content in *C. rudis* bodies. For instance, rutin showed a significant increase in concentration when larvae were fed 10 and 100 mg/g diet, presenting values of 57.30 ± 1.27 and 187.19 ± 1.70 µg/g, respectively. In addition, myricetin also presented a significant increase in concentration from 26.41 ± 2.43 to 105.27 ± 1.33 µg/g at 10 and 100 mg/g, respectively. The amount of gallic acid increased as concentration increased from 10.49 µg/g at 0.1 µg/g to 97.68 µg/g at 100 µg/g (Table 6). Vanillin, ferulic acid, and catechin were found in the bodies of larvae fed a diet supplemented with 100 µg/g of each compound. Quercetin and caffeic acid were not found in larva bodies. Finally, compounds (F_9,80_ = 5995.6, *p* ≤ 0.001), concentrations (F_3,80_ = 2329.6, *p* ≤ 0.001), and their interaction (F_27,80_ = 22,859, *p* ≤ 0.001) had a significant influence on the content of PC in feces of larvae (Table 6). The higher concentration was observed when larvae consumed a diet with vanillin at 100 mg/g concentration (9163.96 ± 10.70 µg/g) and quercetin at 100 mg/g concentration (7870.46 ± 83.39 µg/g). In general, quercetin, vanillin, ferulic acid, caffeic acid, and catechin were eliminated by feces when a high concentration of these compounds was present in the diet (10 or 100 mg/g concentration). Nevertheless, kaempferol, rutin, myricetin, and gallic acid were found in bodies of *C. rudis*, suggesting possible sequestering of these compounds at 10 or 100 mg/g concentration.

## 3. Discussion

Plant domestication is a long-term process during which plants develop several stages until reaching complete domestication [1,2,5]. In this sense, due to the recent domestication history of *U. molinae*, there are few studies about the influence of domestication on PC content and its relationship with the feeding behavior by *C. rudis* [11,13,15,17,20]. For instance, in choice assay, 70% of consumed leaf area in wild ecotypes compared to 40% in cultivated plants was observed, and the data were supported by a no-choice assay where a larger consumed area was in wild plants at 45%, versus 35% in cultivated plants [15]. These data were opposite to what was expected because *C. rudis* larvae preferred to consume the cultivated ecotypes over the parental ecotypes. Later, the effect of the experiment mentioned above and reported by [15] was corrected in [17] by using a common garden to avoid the environmental influence in the experiment. Results of choice and no-choice assays showed the opposite effect in comparison to the previous study [15], supporting the plant domestication defense hypothesis [8]. On the other hand, the choice assay reported in this work indicates a preference in consumption for parental ecotype leaves. However, when ecotype 23-2p was used as parental material (ecotype 17-4o or 66-2o), a decrease in the consumption of parental ecotypes leaves and an increase in the consumption of cultivated ecotypes was observed. These results could suggest that ecotype 23-2p presents a pool of defenses different from other parental ecotypes (ecotypes 19-1o and 22-1o) and that the defense was lost in the offspring. Nevertheless, there was no clear relationship between the total PC concentrations from leaves of the parental or cultivated ecotypes and the performance of the larvae of *C. rudis*.

In relation to PC concentration, the process of domestication in murtilla did not generate a reduction in PC content for all the studied ecotypes. In general, our previous reports contrast with the present results, showing that all cultivated ecotypes presented higher total PC content than ecotypes 19-1p and 23-2p, but ecotype 22-1p had a higher concentration of total PC content, similar to what was reported in [15,17]. In addition, the lowest value of total PC content was observed in ecotype 23-2p. This last result indicates that *C. rudis* had a reduced consumption with ecotype 23-2p, but probably this feeding behavior was not associated with the evaluated PC content because the election test showed that ecotypes 19-1p and 22-1p were preferred over all the offspring regardless of the PC content present in leaves.

Our data suggest that due to the evolutionary history between *C. rudis* and *U. molinae* reported in [15,16,17], the consequences of plant domestication could be modified through this evolutionary process, and larvae will develop a mechanism to tolerate the different PC content present in murtilla leaves. Nevertheless, when the influence of PC was evaluated with an artificial diet, it was possible to establish that the percentage of consumption and mass gain for all PC at any concentration were lower than in control. The reduction in the percentage of consumption and the loss represented by mass gain in larvae did not affect their survival. This could indicate that for *C. rudis* larvae there was no toxic effect of the PC tested. Data of PC from extracts of bodies and feces of *C. rudis* are still surprising due larval excretion, but at the same time the presence of PC inside the bodies could indicate a certain mechanism of detoxification or sequestration [24]. This result suggests that domestication would function in more than one aspect of the chemical defense (other metabolites) involved in the performance of *C*. *rudis* larvae, which will be necessary to study in depth in the future.

Our main findings showed that there was no difference in leaves consumption and mass gain in *C. rudis* larvae when they were fed with leaves of murtilla ecotypes. Moreover, the concentration of PC was significantly higher in cultivated ecotypes mainly when they were obtained from the ecotype 23-2 as parental material. The most abundant compounds in murtilla leaves, either parental or cultivated material, were vanillin, rutin, catechin, and gallic acid.

## 4. Materials and Methods

### 4.1. Plant Material and Insect Collection

Seven ecotypes of *U. molinae* plants were used to perform the experiments. Plants were ordered into 2 groups: (1) parental ecotypes (P), corresponding to ecotypes 19-1p, 22-1p, and 23-2p, and (2) cultivated ecotypes (O), corresponding to the offspring among the parental ecotypes: 10-1o, 16-16o, 17-4o, and 66-2o (Table 7). Cultivated ecotypes were compared with their corresponding parental ecotypes. All ecotypes were kept at 25 °C in the Laboratory of Chemical Ecology at the Universidad de La Frontera, Temuco, Chile. Ten leaves per plant were collected according to the cardinal points (N, E, S, W) and used immediately in preference assays [15]. For the phenolic extraction procedure (see below), we collected healthy whole leaves in the same way, but they were deposited in plastic bags and stored at −20 °C until their use for chemical analysis. *C. rudis* larvae were collected manually from natural grassland in Temuco, Región de La Araucanía, Chile, in late summer. Larvae of the last instars (≈35 mm) were used to perform the experiments, and they were deprived of food 12 h beforehand.

### 4.2. No-Choice Assay

A single *C. rudis* larva was placed in a Petri dish (94 mm in diameter by 16 mm high) containing either a cultivated or parental ecotypes healthy and whole *U. molinae* leaf from top of the plant. After 48 h, the bioassay was completed, and the mass gain and consumption were evaluated [25]. Fresh larval mass was obtained prior to the bioassay and after 48 h, and larvae were weighted at the end of the experiments, with the variation of mass was expressed as percentage [26]. Finally, consumption was measured in square centimeters by scanning each leaf and then measuring the area consumed using ImageJ 1.42j software (National Institutes of Health, Bethesda, Maryland, USA) [27]. Leaves were replaced after 24 h, and the mean of both leaves (24 h and 48 h) were used to calculate the consumption as percentage (*n* = 10).

### 4.3. Choice Assay

One cultivated and one parental ecotypes leaf of *U. molinae* were placed equidistantly in a Petri dish. A single larva was placed equidistant from the leaves in the Petri dish and allowed to feed for 48 h. The mass gain and consumption were evaluated at the end of 48 h as in the no-choice assay [28]. Leaves were replaced after 24 h, and the mean of both leaves (24 h and 48 h) was used to calculate the consumption as percentage (*n* = 10).

### 4.4. Phenolic Compound Extraction

A total of 21 samples from leaves of all 7 ecotypes, cultivated and parental ecotype plants, were collected from the 4 cardinal points at the same height. Samples were placed in an oven at 60 °C for 48 h. Then, they were milled in a grinder, and 5 g of sample was placed in a flask, to which a mixture of methanol and water (1:1) was added. A solvent-to-solid ratio of 5:1 was applied. The flasks were placed in a magnetic stirrer for 20 min at 30 °C and 170 rpm. After this, samples were filtered in the dark through Whatman number 1 filter paper (Whatman International Ltd., Maidstone, UK). The filtrate was concentrated in a rotary evaporator at 45 °C and lyophilized for 12 h. Finally, each sample was suspended in 5 mL of methanol and left for 5 min in a Branson 3510 Ultrasonic Cleaner. Samples were stored at 20 °C in amber flasks until they were used in high-performance liquid chromatography (HPLC) analysis [15,17,18].

### 4.5. Dose–Response Experiment

An artificial diet was prepared according to the methodology proposed in [29] (Table 8). For 1000 mg of diet, doses of 0.01, 0.1, 1, and 10% of each PC standards (caffeic acid, ferulic acid, gallic acid, catechin, kaempferol, myricetin, quercetin, rutin, and vanillin) were placed into Petri dishes to obtain concentrations of 0.1, 1, 10, and 100 mg/g. A single larva was weighed and placed in each Petri dish. The mass gain, consumption, and survival were evaluated at 24, 48, 72, and 96 h. At the same time, the survival of larvae was measured as a binary categorical variable (alive or dead), and all alive insects were censored at the end of the experiment (96 h). The artificial diet was changed every 2 days to avoid contamination (bacteria and fungus). In addition, at the end of the assay, larvae and feces were collected and stored at −20 °C to determine the PC content by HPLC (*n* = 10).

### 4.6. Extraction of Bodies and Feces

Individual larvae were dissected, and guts were removed to avoid any influence on chemical analysis. Bodies of *C. rudis* larvae and their feces were dried in an oven at 45 °C for 72 h. Then, samples were milled, and extraction was performed according to the methodology proposed in [24]. Briefly, 25 mg of powder was extracted with 2 mL of methanol/water/formic acid (70:29.5:0.5 *v/v/v*) and centrifuged at 16,000 rpm for 3 min. After this, supernatant was collected and filtered in a 0.22 mm membrane before being injected into an HPLC.

### 4.7. HPLC-DAD Analysis

Methanolic extracts obtained from murtilla leaves and bodies and feces of *C. rudis* were filtered through a 0.22 mm membrane and analyzed by HPLC. For the analysis, 10 µL of each sample was injected into a Shimadzu HPLC (Model LC-20A Prominence, Kyoto, Japan) equipped with a C-18 column (300 mm × 4.6 mm I.D., particle size 5 mm) maintained at 40 °C. The analysis was performed using a linear solvent gradient consisting of 1% formic acid (A) and acetonitrile (B) as follows: 0–50 min, 90% A/10% B; 50–60 min, 45% A/55% B; 60–65 min, 90% A/10% B at a flow rate of 1 mL/min. Gallic acid, catechin, kaempferol, myricetin, rutin, and quercetin were monitored at 262 nm, and caffeic acid, ferulic acid, and vanillin were monitored at 310 nm; UV spectra from 190 at 700 nm were used for peak characterization. The identification of PC was based on the peak retention time in comparison with pure standard. To construct a calibration curve for each compound, we dissolved standard solutions in methanol at 1000 mg/L. The stock solution of each standard was used to prepare serial concentrations between 0.05 and 500 mg/L. All standards were stored at 4 °C before being injected into the HPLC. To determine the limits of detection (LOD) and quantification (LOQ), we diluted the stock solution of each standard in MeOH to provide serial dilutions. Each solution was injected into the HPLC device until a 3-r signal-to-noise (S/N) ratio for the LOD and 10-r for the LOQ of each polyphenolic compound was obtained [30].

### 4.8. Statistical Analysis

Statistix 10 statistical software (Tallahassee, FL, USA) was used to perform the analysis. For choice and no-choice assays, the mass gain and consumption in *C. rudis* larvae in relation to the ecotype effect underwent one-way analysis of variance (ANOVA) followed by Tukey’s test. In addition, in choice assays, Student’s *t*-test was performed to analyze the difference between cultivated and parental ecotypes in relation to consumption. The principal effects of PC, ecotypes, and their interaction on the phenolic concentration was also analyzed using ANOVA followed by Tukey’s test. To analyze the mass gain of larvae and consumption in the dose-response experiment over time at several doses, we used ANOVA followed by Tukey’s test, and a regression was carried out for determining the variable explanatory related to mass gain or consumption. In addition, Student’s *t*-test was performed to compare the percentage of consumption and mass gain of the control treatment with each PC at the respective time. The survival of larvae was analyzed by a Kaplan–Meier survival analysis using log-rank test. For multiple comparisons, the Holm-Sidak test was performed. Finally, the concentration of PC in bodies and feces of *C. rudis* were analyzed using ANOVA followed by Tukey’s test. Data were natural-log transformed to meet the assumption of normality and homogeneity of variance. Percentages were arcsin square root-transformed.

## 5. Conclusions

In conclusion, there was no relationship between PC and the plant domestication process in murtilla. In addition, a higher concentration of PC was found in leaves of the cultivated plants. Thus, there was no clear reduction in chemical defenses due to domestication. Further analysis of domestication of murtilla will be addressed in future, increasing the knowledge of trade-off between growth rate and chemical defense and when this trade-off significantly reduces the chemical defenses associated with the main insect pests in murtilla plants.

## Figures and Tables

**Table 1:** Consumption of leaves and mass gain of *Chilesia rudis* for each comparison run in a choice assay. Values of *p* < 0.05 indicate significant differences according to Student’s *t*-test for consumption and for mass gain an ANOVA followed by Tukey’s test.

Parental Ecotypes		Cultivated Ecotype	Leaf Consumption (%)	*p*	Mass Gain (%)	*p*
19-1p	×	10-1o	31.6 ± 8.0 vs. 16.2 ± 6.6	0.31	−8.0 ± 6.9	0.42
19-1p	×	16-16o	29.0 ± 5.4 vs. 6.0 ± 4.0	≤0.001	−1.9 ± 0.5	
19-1p	×	66-2o	47.3 ± 6.0 vs. 1.2 ± 0.4	≤0.001	−1.5 ± 0.9	
22-1p	×	10-1o	18.4 ± 3.7 vs. 6.6 ± 3.4	≤0.001	1.8 ± 0.9	0.09
22-1p	×	16-16o	17.7 ± 6.4 vs. 3.2 ± 1.2	≤0.001	1.2 ± 1.0	
22-1p	×	17-4o	24.5 ± 7.8 vs. 9.8 ± 3.2	≤0.001	−8.0 ± 6.9	
23-2p	×	17-4o	9.1 ± 3.3 vs. 3.2 ± 1.6	≤0.001	−1.3 ± 3.8	0.15
23-2p	×	66-2o	10.6 ± 4.4 vs. 4.8 ± 1.8	0.07	6.7 ± 4.9	

**Table 2:** Content of phenolic compounds found in *Ugni molinae* leaves in relation to domestication degree. Lowercase letters indicate significant differences of compound and uppercase letters indicate significant differences of total concentration according to ANOVA and Tukey’s test (*p* ≤ 0.05).

	Parental Ecotypes/Cultivated Ecotype						
Compound (µg/g Dry Mass)	Parental Ecotypes	Parental Ecotypes	Parental Ecotypes	Cultivated	Cultivated	Cultivated	Cultivated
				22-1p × 19-1p	19-1p × 22-1p	23-2p × 22-1p	23-2p × 19-1p
	19-1p	22-1p	23-2p	10-1o	16-16o	17-4o	66-2o
Caffeic acid	1.4 ± 1.0hi	51.9 ± 29.4f	2.7 ± 0.5h	5.0 ± 2.3h	7.6 ± 4.3h	1.8 ± 0.1h	3.6 ± 1.4h
Ferulic acid	8.4 ± 4.1h	31.1 ± 7.3g	1.2 ± 0.1h	32.9 ± 4.0g	27.9 ± 3.4g	2.0 ± 1.0h	3.8 ± 1.0h
Gallic acid	12.0 ± 10.2gh	73.6 ± 32.9f	18.1 ±7.3gh	34.1 ± 15.5g	64.4 ± 38.7f	12.6 ± 0.7g	59.7 ± 16.1f
Catechin	15.7 ± 5.1g	190.7 ± 82.8cd	3.8 ± 1.4h	101.1 ± 26.4f	41.5 ± 24.0g	28.8 ± 26.1g	164.9 ± 82.4ef
Kaempferol	0.1 ± 0.0i	0.3 ± 0.0i	NDi	0.1 ± 0.0i	0.1 ± 0.0i	0.1 ± 0.0i	1.0 ± 0.0i
Myricetin	8.2 ± 4.8h	15.8 ± 2.6g	3.6 ± 1.8h	17.1 ± 6.8g	18.8 ± 2.5g	7.2 ± 3.9h	28.3 ± 7.4g
Quercetin	1.0 ± 0.8hi	10.7 ± 3.1g	0.5 ± 0.2h	5.1 ± 2.8h	5.7 ± 2.2h	2.1 ± 0.5h	4.5 ± 0.6h
Rutin	12.0 ± 8.1gh	115.3 ± 14.4ef	5.5 ± 2.6h	133.7 ± 40.0ef	106.5 ± 20.4f	21.5 ± 5.2g	63.3 ± 13.3f
Vanillin	201.9 ± 99.0de	1537.9 ± 543.7a	97.0 ± 57.0f	1571.0 ± 697.1a	883.7 ± 183.6bc	994.8 ± 580.8bc	1420.6 ± 701.9ab
Total	260.7 ± 14.7D	2027.3 ± 79.5A	132.4 ± 7.8E	1900.1 ± 88.3AB	1156.2 ± 31.0C	1070.9 ± 68.7C	1179.7 ± 91.5C

ND, not detected.

**Table 3:** Percentage of diet consumption by *C. rudis* at several doses and different times (h). Letters indicate significant differences according to ANOVA followed by Tukey’s test. ANOVA was performed for each individual compound in relation to dose and time. * Significant differences in relation to control treatment according to Student’s *t*-test for respective time (h) and concentration.

Time (h)	Concentration (mg of Compound/g of Diet)	Control (µg/g)	Quercetin (µg/g)	Kaempferol (µg/g)	Myricetin (µg/g)	Rutin (µg/g)	Vanillin (µg/g)	Catechin (µg/g)	Caffeic Acid (µg/g)	Gallic Acid (µg/g)	Ferulic Acid (µg/g)
24	0.1	50.0 ± 16.6	35.5 ± 12.5bcd	63.0 ± 15.2	5.0 ± 1.0 *	45.0 ± 12.7	28.0 ± 12.0 *	40.5 ± 10.0	33.5 ± 3.0	18.0 ± 6.7b	41.5 ± 15.3
	1	70.0 ± 15.2	42.0 ± 15.8bcd *	54.0 ± 13.7	42.0 ± 15.1 *	70.0 ± 13.4	15.5 ± 9.4 *	54.0 ± 12.7	35.0 ± 3.6 *	16.5 ± 5.6b *	47.5 ± 12.6 *
	10	53.0 ± 7.6	24.0 ± 12.6bcd *	55.0 ± 12.5	41.0 ± 14.0	54.0 ± 13.7	11.5 ± 3.9 *	40.0 ± 10.9	40.0 ± 5.2	22.2 ± 7.6ab *	51.0 ± 6.9
	100	42.0 ± 10.4	14.5 ± 9.5bcd *	25.0 ± 5.5	50.5 ± 11.5	55.0 ± 13.3	7.5 ± 0.8 *	24.5 ± 7.4 *	32.0 ± 4.1	24.5 ± 6.3ab *	33.5 ± 5.5
48	0.1	-	100.0 ± 0.0a *	13.0 ± 3.6 *	32.0 ± 8.3	35.7 ± 12.3	49.5 ± 14.0	32.5 ± 9.3	39.0 ± 8.0	46.5 ± 11.3a	64.5 ± 5.0
	1	-	52.5± 15.8b	7.0 ± 1.1 *	51.0 ± 11.8	56.0 ± 12.7	33.5 ± 10.6 *	62.0 ± 11.8	37.0 ± 6.7 *	17.0 ± 6.4b *	38.5 ± 11.3 *
	10	-	49.7 ± 15.1bc	10.2 ± 0.9 *	55.0 ± 8.7	41.5 ± 10.2	20.0 ± 6.2 *	40.0 ± 10.9	37.0 ± 7.8 *	11.0 ± 4.9b *	28.5 ± 9.9 *
	100	-	56.1 ± 14.0bcd	3.6 ± 0.7 *	39.5 ± 11.6	79.0 ± 7.4*	9.0 ± 1.1 *	24.5 ± 7.4	39.0 ± 5.6	20.0 ± 7.5ab *	22.2 ± 6.9 *
72	0.1	-	16.4 ± 7.1bcd *	9.0 ± 1.4 *	22.5 ± 6.6 *	47.0 ± 8.9	14.0 ± 6.4 *	8.5 ± 1.8 *	23.0 ± 4.0 *	18.5 ± 4.2b *	28.5 ± 6.4 *
	1	-	9.5 ± 4.5cd *	5.1 ± 1.2 *	63.5 ± 8.3	50.4 ± 13.8	12.0 ± 4.0 *	9.0 ± 1.7 *	16.5 ± 7.3 *	21.0 ± 3.7ab *	26.5 ± 6.1 *
	10	-	5.5 ± 0.5d *	12.2 ± 5.0 *	41.5 ± 10.7	34.0 ± 11.6	18.7 ± 5.3 *	11.0 ± 3.9 *	19.5 ± 9.1 *	18.0 ± 4.7b *	28.0 ± 6.4 *
	100	-	28.5 ± 12.7bcd	7.5 ± 0.8 *	36.0 ± 12.1	50.0 ± 13.6	7.9 ± 1.4 *	4.5 ± 2.1 *	7.8 ± 1.8 *	14.0 ± 8.1b *	22.5 ± 6.9 *
96	0.1	-	10.5 ± 2.4cd *	2.9 ± 0.4 *	37.5 ± 5.6	41.5 ± 11.3	4.0 ± 1.7 *	7.5 ± 4.8 *	6.0 ± 1.2 *	5.0 ± 1.2b *	22.5 ± 5.2 *
	1	-	8.0 ± 1.6cd *	5.5 ± 0.5 *	51.0 ± 11.8	34.0 ± 10.6 *	2.5 ± 1.5 *	3.0 ± 2.0 *	4.5 ± 1.1 *	3.5 ± 1.3b *	18.0 ± 5.3 *
	10	-	11.0 ± 2.0d *	4.7 ± 1.3 *	41.5 ± 10.7	60.5 ± 12.2	7.5 ± 2.0 *	3.0 ± 0.8 *	5.0 ± 0.7 *	1.5 ± 1.5b *	14.5 ± 4.9 *
	100	-	6.0 ± 4.9d *	5.1 ± 1.4 *	48.5 ± 11.1	46.7 ± 13.2	5.5 ± 1.1 *	2.5 ± 1.1 *	5.5 ± 1.1 *	0.5 ± 0.5b *	17.0 ± 3.1 *

**Table 4:** Percentage of mass gain at several doses and times (h) for *C. rudis*. Letters indicate significant differences according to ANOVA followed by Tukey’s test. ANOVA was performed for each compound in relation to dose and time. * Significant differences in relation to control treatment according to Student’s *t*-test for respective time (h) and concentration.

Time (h)	Concentration (mg of Compound/g of Diet)	Control (µg/g)	Quercetin (µg/g)	Kaempferol (µg/g)	Myricetin (µg/g)	Rutin (µg/g)	Vanillin (µg/g)	Catechin (µg/g)	Caffeic Acid (µg/g)	Gallic Acid (µg/g)	Ferulic Acid (µg/g)
24	0.1	25.9 ± 11.0	12.8 ± 4.9b *	32.7 ± 2.4a *	25.4 ± 1.8b	62.3 ± 2.4a *	18.8 ± 6.0a	22.0 ± 2.1a	24.9 ± 4.2a	13.3 ± 2.8a *	13.5 ± 3.2ab *
	1	25.4 ± 1.3	23.8 ± 8.9a	21.3 ± 1.0b	33.2 ± 2.4a *	47.8 ± 2.6ba *	18.6 ± 6.3a	22.6 ± 3.5a	14.0 ± 1.3b *	14.1 ± 2.9a *	16.2 ± 4.0a *
	10	24.5 ± 2.2	21.4 ± 4.1a	18.4 ± 0.4b *	33.2 ± 0.3a *	47.9 ± 0.3b *	18.8 ± 4.5a *	20.0 ± 4.4a	14.9 ± 2.6b *	15.0 ± 4.4a *	16.5 ± 0.8a *
	100	28.4 ± 3.6	10.3 ± 1.6b *	18.7 ± 0.7b *	33.4 ± 0.7a	48.2 ± 0.7b *	11.4 ± 2.8b *	22.1 ± 2.5a	10.9 ± 1.6b *	13.0 ± 2.9a *	18.4 ± 2.5a *
48	0.1	-	6.1 ± 0.9b *	25.4 ± 1.2b	22.2 ± 2.7b	33.6 ± 2.7c *	12.2 ± 2.3b *	6.0 ± 0.4b *	18.8 ± 3.2b *	8.9 ± 1.6b *	14.8 ± 3.2ab *
	1	-	7.3 ± 1.2b *	24.3 ± 0.8b	14.4 ± 4.8c *	24.1 ± 4.8d	9.0 ± 1.8b *	5.2 ± 0.5b *	12.1 ± 1.6b *	7.4 ± 1.2b *	13.0 ± 2.4ab *
	10	-	7.0 ± 1.2b *	22.1 ± 0.3b	16.9 ± 0.3c *	27.2 ± 0.7d	9.3 ± 2.5b *	7.6 ± 1.0b *	5.4 ± 0.9c *	7.2 ± 1.3b *	7.3 ± 0.3c *
	100	-	6.9 ± 1.0b *	22.3 ± 0.7b	17.1 ± 0.7c *	25.8 ± 0.6d	9.9 ± 2.5b *	7.2 ± 0.7b *	5.9 ± 0.9c *	6.8 ± 0.9b *	7.5 ± 0.7c *
72	0.1	-	6.5 ± 1.3b *	10.1 ± 2.4c *	34.9 ± 2.4a *	29.7 ± 2.4d	18.6 ± 10.2a *	-13.5 ± 1.1c *	8.6 ± 1.3c *	4.6 ± 1.0b *	14.0 ± 0.6ab *
	1	-	6.2 ± 1.1b *	−6.6 ± 4.8d *	18.1 ± 4.8c *	12.9 ± 4.8e *	10.4 ± 5.3b *	−11.8 ± 1.4c *	6.1 ± 1.1c *	8.8 ± 2.3b *	15.4 ± 2.2ab *
	10	-	3.3 ± 0.8c *	−4.2 ± 0.3d *	20.6 ± 0.3b	15.4 ± 0.3e *	1.2 ± 3.1c *	−18.0 ± 3.5c *	7.9 ± 1.0c *	1.0 ± 0.6c *	11.0 ± 0.3b *
	100	-	3.9 ± 0.8c *	−3.9 ± 0.7d *	20.8 ± 0.7b *	15.6 ± 0.7e *	0.6 ± 1.2d *	−16.4 ± 2.2c *	9.6 ± 1.6bc *	−0.8 ± 2.2c *	11.2 ± 0.7b *
96	0.1	-	−2.1 ± 3.0d *	−6.1 ± 2.4d *	11.6 ± 1.2c *	23.4 ± 2.4d	−4.5 ± 2.3f *	−0.2 ± 2.0c *	5.6 ± 0.9c *	−3.8 ± 1.8d *	9.0 ± 2.4c *
	1	-	−0.6 ± 9.6d *	−10.9 ± 1.3e *	9.0 ± 1.6c *	6.6 ± 4.8f *	−0.8 ± 4.1e *	2.4 ± 2.3bc *	4.6 ± 1.2c *	1.9 ± 2.2c *	−7.7 ± 4.8d *
	10	-	−1.3 ± 1.9d *	−12.5 ± 1.2e *	7.3 ± 2.1c *	9.1 ± 0.3f *	−2.0 ± 2.9f *	−14.7 ± 12.2c *	3.2 ± 1.0c *	−2.1 ± 5.0d *	−5.3 ± 0.3d *
	100	-	−3.8 ± 1.4d *	−13.2 ± 1.1e *	3.5 ± 0.4d *	9.3 ± 0.7f *	−4.4 ± 1.1f *	−6.9 ± 5.0c *	−2.5 ± 3.7d *	−0.1 ± 1.7c	−5.0 ± 0.7d *

**Table 5:** Regression analysis for leaves consumption and mass gain of *Chilesia rudis* in dose-response experiments. Italics number mean significant differences.

	Leaves Consumption			Mass Gain		
	Predictor *p*-Value for Hours	Predictor *p*-Value for Concentrations	Adjusted *R^2^*	Predictor *p*-Value for Hours	Predictor *p*-Value for Concentrations	Adjusted *R^2^*
Quercetin	0.009	0.510	0.044	0.568	0.005	0.037
Kaempferol	0.015	0.003	0.073	0.001	0.001	0.234
Myricetin	0.010	0.867	0.028	0.001	0.001	0.234
Rutin	0.800	0.001	0.078	0.001	0.001	0.234
Vanillin	0.001	0.001	0.305	0.872	0.117	0.003
Catechin	0.015	0.001	0.083	0.243	0.001	0.299
Caffeic acid	0.043	0.160	0.025	0.001	0.392	0.053
Gallic acid	0.001	0.001	0.200	0.117	0.001	0.088
Ferulic acid	0.001	0.352	0.059	0.001	0.001	0.234

**Table 6:** Concentration of compounds in bodies and feces of *C. rudis.* Letters indicate significant differences according to ANOVA and Tukey’s test separately for larvae and feces (*p* ≤ 0.05).

	Concentration (mg of Compound/g of Diet)	Quercetin(µg/g)	Vanillin (µg/g)	Kaempferol (µg/g)	Rutin (µg/g)	Myricetin (µg/g)	Ferulic Acid (µg/g)	Caffeic Acid (µg/g)	Gallic Acid (µg/g)	Catechin (µg/g)	Control (µg/g)
**Larvae**	0.1	ND	ND	ND	ND	ND	ND	ND	10.4 ± 0.0h	ND	ND
	1	ND	ND	ND	ND	ND	ND	ND	10.1 ± 0.1h	ND	ND
	10	ND	ND	2.9 ± 0.1i	57.3 ± 1.2d	26.4 ± 2.4f	ND	ND	14.0 ± 0.1g	ND	ND
	100	ND	134.8 ± 2.8b	14.4 ± 0.1g	187.1 ± 1.7a	105.2 ± 1.3c	39.0 ± 0.8e	ND	97.6 ± 1.5c	9.8 ± 1.5h	ND
**Feces**	0.1	ND	ND	ND	ND	ND	12.0 ± 0.1i	ND	ND	ND	ND
	1	ND	ND	ND	ND	ND	16.1 ± 0.1d	ND	ND	ND	ND
	10	307.6 ± 6.7c	12.5 ± 0.0i	ND	ND	ND	112.5 ± 1.8h	ND	24.7 ± 0.1g	ND	ND
	100	7870.4 ± 83.3b	9163.9 ± 10.7a	0.5 ± 0.0j	26.9 ± 0.5f	33.7 ± 0.6f	11.7 ± 0.1d	28.5 ± 0.0f	74.5 ± 1.2e	115.5 ± 1.5d	ND

ND, not detected.

**Table 7:** Origin of cultivated ecotypes and their corresponding parental ecotypes.

Cultivated Ecotypes (Offspring)	Parental Ecotypes
10-1o	22-1p × 19-1p
16-16o	19-1p × 22-1p
17-4o	23-2p × 22-1p
66-2o	23-2p × 19-1p

**Table 8:** Composition of artificial diet used in dose–response experiment with *Chilesia rudis*.

Ingredient	Amount
Green peas	75 g
Yeast	17.5 g
Ascorbic acid	3.5 g
Boiled egg yolk	7.5 g
Sorbic acid	0.5 g
Formaldehyde 37%	4.05 mL
Agar	15 g
Water	376.95 mL

## Data Availability

Data sharing is not applicable to this article.

## Sample Availability

Samples of the compounds are not available from the authors.
